# Reverberant optical coherence elastography using 3D-printed randomly distributed scatterers: elasticity mapping of hydrogels in culture dishes

**DOI:** 10.1117/1.JBO.30.12.124507

**Published:** 2025-10-07

**Authors:** Hao Xu, FanLei Yang, Ting Liang, Wen Zhang, JianQiang Mo, ZongPing Luo

**Affiliations:** a The First Affiliated Hospital of Soochow University, Department of Orthopedics, Suzhou, China; b Soochow University, Orthopedic Institute, Medical College, Suzhou, China

**Keywords:** optical coherence tomography, reverberant wave elastography, 3D-printed randomly distributed scatterers, hydrogel, cell culture dish

## Abstract

**Significance:**

Accurate estimation of hydrogel phantom elasticity in 3D cell culture systems provides valuable insights into cellular responses to various mechanical stimuli. Although reverberant wave elastography has been applied to measure hydrogel elasticity in 3D cell cultures using multi-point loading, achieving a high-quality reverberant displacement field remains critical for accurate reverberant wave elastography.

**Aim:**

We develop an innovative approach using 3D-printed randomly distributed scatterers to improve displacement field quality in reverberant wave elastography, inspired by scattering-coded architectured boundaries in object localization.

**Approach:**

Numerical simulations were performed to analyze the reverberant displacement fields under various loading conditions. The results were compared to determine the optimal loading configuration to enhance the reverberation level of the displacement field. Subsequently, both numerical and experimental reverberant wave elastography were carried out to validate the elasticity measurement with 3D-printed randomly distributed scatterers.

**Results:**

The comparison of reverberant displacement patterns under various loading conditions revealed that the displacement pattern under circular loading with 64 scatterers most closely approximated a diffuse wave field, exhibiting both spatial uniformity and directional isotropy. Numerical reverberant wave elastography was subsequently performed, successfully demonstrating its capability for elasticity measurements. Furthermore, the shear wave speeds obtained through optical coherence elastography showed good agreement with shear rheometry measurements.

**Conclusions:**

The developed 3D-printed randomly distributed scatterers successfully enhanced the quality of the reverberant displacement field for reverberant wave elastography. Our approach presents a novel and promising tool for quantifying tissue elasticity in reverberant wave elastography applications.

## 1 Introduction

As synthetic extracellular matrices, hydrogels have emerged as indispensable biomaterial platforms in tissue engineering, enabling reconstruction of native tissue structure and function.^1^^–^^3^ Their mechanical properties are particularly critical, serving as key regulators of cellular phenotype and tissue development.^4^^,^^5^ Moreover, the elasticity of hydrogels can undergo continuous modifications due to various processes such as swelling, enzymatic degradation or crosslinking, and particularly cell-mediated remodeling.^6^^,^^7^ As a result, there is a clear need for a method that can track the dynamic changes in hydrogels’ elasticity over time.

Common material testing techniques, including tension, compression, indentation, and atomic force microscopy,^8^^,^^9^ rely on physical contact, which may introduce contamination and sample damage. Consequently, their application to hydrogel elasticity measurement is limited.^10^ Wave-based elastography provides a non-invasive approach to measure hydrogel elasticity by using optical coherence elastography,^11^ ultrasound shear wave elastography (SWE),^12^^–^^14^ and magnetic resonance elastography (MRE).^15^^,^^16^ For instance, transient wave-based elastography has been employed in the assessment of hydrogel elasticity.^11^ However, to ensure proper propagation of the mechanical wave across the image plane from the loading point, a complex focusing alignment procedure was utilized. To overcome the need for complex focusing alignment procedures, directional filters were employed to separate elementary shear waves that constitute a complex displacement field. Subsequently, this approach facilitated measurement of mechanical properties in gelatin phantoms within cell culture plates.^11^^,^^17^ It should be noted that the wave reflections at boundaries are inevitable during hydrogel elasticity measurement in 3D cell cultures. Therefore, estimating the wave speed from the complex wave fields is preferable. Accordingly, a novel method inspired by seismic noise correlation^18^ has been developed to extract tissue elasticity using cross-correlation and auto-correlation techniques.^19^ This approach leverages complex wave fields generated by the interaction of multiple waves traveling from all possible directions. Based on broadband noise-like wave fields and cross-correlation techniques, passive elastography was developed to estimate the local elasticity in the medium and has already been used for several organs such as the liver, the thyroid, or the brain.^20^ Up to now, the passive elastography has been demonstrated with ultrasound,^21^^–^^25^ optical coherence tomography (OCT),^26^^,^^27^ digital holography,^28^^–^^30^ and MRI.^31^ Unlike passive elastography, reverberant elastography employs active sources vibrating at a single frequency to generate a narrowband diffuse field. The shear wave speed is then derived from the spatial autocorrelation function.^32^^–^^36^

Recently, hydrogel elasticity measurement in the slice plane was performed in culture disks according to the reverberant wave theory,^35^ without the focusing alignment and directional filters. The validity of reverberant wave theory requires that the displacement field satisfy the conditions of a diffuse wave field, exhibiting both spatial uniformity and directional isotropy. Hence, improving the quality of the reverberant displacement field is significant for reverberant wave elastography. Previous studies have introduced various excitation methods to generate reverberant displacement fields, including an array of actuators,^32^ a custom-made portable tri-fold futon with embedded quad resonators,^37^ a multi-pronged ring actuated by a piezoelectric bender,^33^ eight-point loading in the bottom of the culture disk,^35^ and multifocal acoustic radiation force.^34^^,^^36^ Despite their complexity, all these methods provide multi-point loading. Recently, a scattering-coded architectural boundary composed of randomly distributed scatterers was adopted in object localization.^38^ The multiple scattering effects introduced by this architectured boundary increase the complexity of the scattered displacement fields, enabling a highly uncorrelated scattering coding of surface waves.

This study investigates the application of reverberant wave elastography with a 3D-printed randomly distributed scatterer for mechanical behavior characterization of hydrogel biomaterials in culture dishes. First, the displacement fields by different loading conditions are compared to find the best condition to enhance the reverberation level of the displacement field, including the circle loading, 8- and 16-point loading, and circle loading with 3D-printed randomly distributed scatterers (8, 16, 32, and 64 scatterers). Then, finite element analysis is performed to validate the measurement of shear wave speed by the current reverberant wave elastography method. Finally, the proposed method is used to measure the shear wave speed of hydrogels with different concentrations using OCT, and the shear rheology tests are performed for validation.

## 2 Materials and Methods

### 2.1 Fabrication of 3D-Printed Randomly Distributed Scatterers and Homogeneous Phantoms

The 3D-printed randomly distributed scatterers consist of 64 scatterers positioned at distances R1 (14 mm) and R2 (15 mm) from the center. All scatterers share a diameter of 0.7 mm and a thickness of 5 mm. The angle θ between adjacent scatterers is a randomly assigned variable governing their positions.

**Fig. 1:**
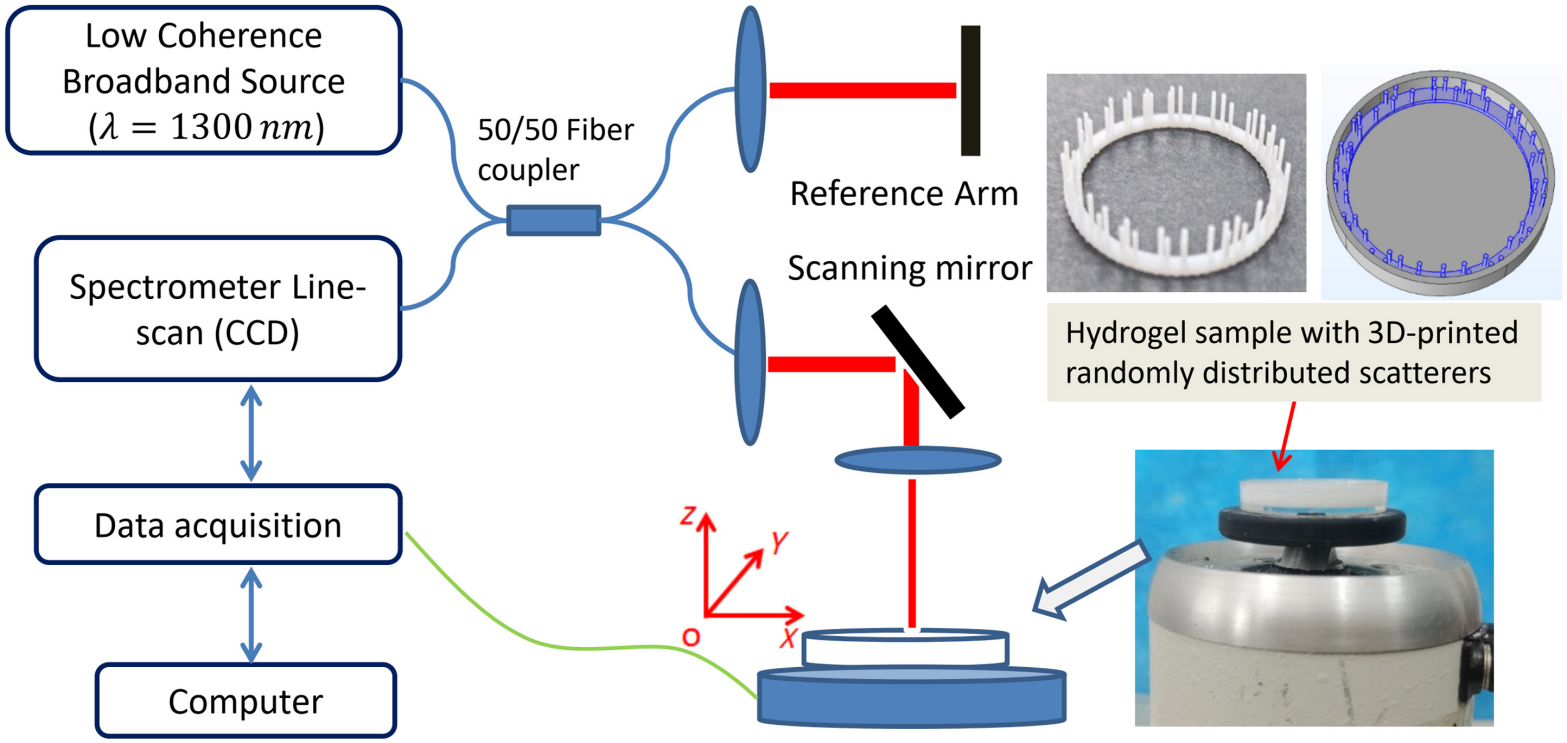
Experimental setup for elasticity measurement of the hydrogel in culture disks using reverberant optical coherence elastography. The 3D-printed randomly distributed scatterers were adopted to generate the reverberant displacement field in the phantom. The scatterer diameter and thickness are 0.7 and 5 mm, and the phantom thickness is 5 mm.

The hydrogel phantom was prepared to perform reverberant wave elastography. The hydrogel concentrations used were 4% v/v and 5% v/v (GelMAEFL-GM-60, Engineering For Life, China). Each hydrogel phantom was created by combining hydrogel powder with 10 mL of photoinitiator solution and 0.05 g of titanium dioxide as optical scatterers. For the reverberant wave elastography experiments, the mixed hydrogel solution was poured into culture dishes (diameter, 34 mm; thickness, 6 mm) containing 3D-printed randomly distributed scatterers (Fig. 1). The bottom of the culture dishes is a hollow cylinder. Similarly, the same hydrogel solution was filled into a cylindrical container (diameter, 20 mm; height, 3 mm) for shear rheology tests. The hydrogel samples were cured using UV light irradiation for a duration of 40 s.

### 2.2 Elasticity Measurements Based on Reverberant Wave Elastography

**Fig. 2:**
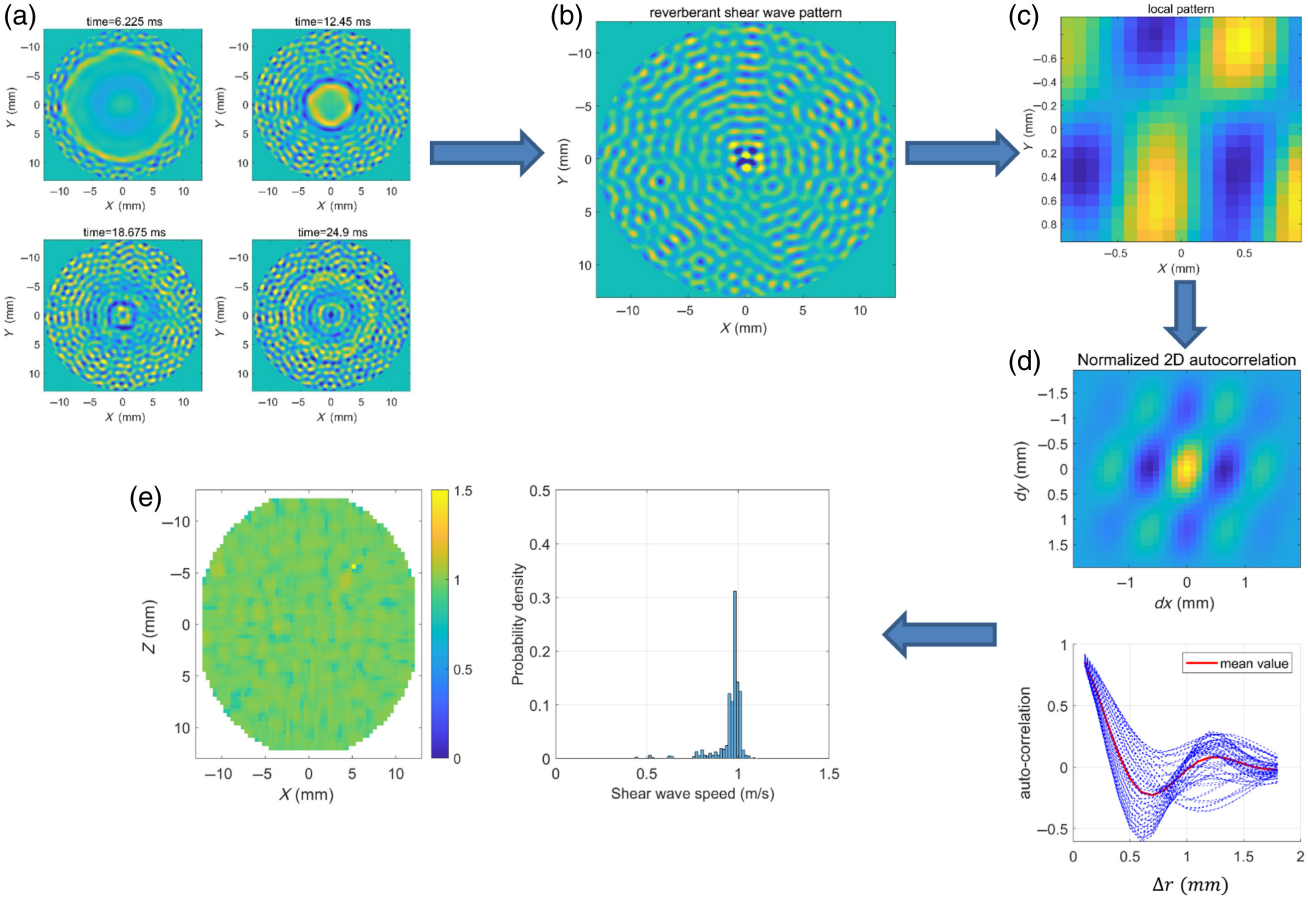
(a) Time-domain displacement field at the sample surface. (b) The reverberant wave pattern at frequency 800 Hz. (c) The local reverberant wave pattern, the window size is set to be 1.5 times the wavelength. (d) Real part of normalized 2D auto-correlation map of the local reverberant shear wave pattern, auto-correlation curves (N=72 curves) taken along radial cuts of the 2D auto-correlation map, and its mean value. (e) Shear wave speed map and probability density. The theoretical shear wave speed in the numerical simulation is 1m/s, and the loading frequency is 800 Hz.

The reverberant field is defined as the superposition of plane waves and can be utilized for estimating the shear wave speed in the medium. The flowchart (Fig. 2) illustrates the complete process of shear wave speed estimation based on the reverberant waves theory.

First, the time-domain displacement field in the sample surface [Fig. 2(a)] was obtained from numerical simulations or experimental tests. Then, a temporal Fourier transform was applied to the displacement field. The magnitude and phase were extracted to create a 2D displacement matrix (reverberant wave pattern) at loading frequency f. In addition, to remove compressional waves and reduce noise, an additional 2D bandpass filter in the spatial frequency domain was applied to the 2D displacement matrix in all directions. The low and high cutoff spatial frequencies are set at $k_{l}=2\pi f_{v}/c_{l}$ and $k_{h}=2\pi f_{v}/c_{h}$, respectively, where $c_{l}$ and $c_{h}$ are two chosen low and high shear wave speeds, respectively. In the current study, $c_{l}$ and $c_{h}$ are 0.2  m/s and 3  m/s, respectively. Now, the reverberant wave pattern was obtained for the shear wave speed estimation [Fig. 2(b)].

Second, the 2D auto-correlation of any local reverberant wave pattern [Fig. 2(c)] was calculated. The auto-correlation curves (N=72 curves) taken along radial cuts of the 2D auto-correlation map covering 360 deg are shown in Fig. 2(d). Then, the average auto-correlation curves $B_{V_{z}V_{z}}(\Delta r)$ was obtained, where Δr is the correlation distance. In addition, the spatial normalized 2D auto-correlation along the r-axis can be described as^39^

$$B_{V_{z}V_{z}}(\Delta r)=j_{0}(\frac{2\pi f}{C_{R}}\Delta r),$$ (1)

where $j_{0}$ is the Bessel function of zero order, f is the loading frequency, and $C_{R}$ is the Rayleigh wave speed. The average auto-correlation curve was fitted to Eq. (1) using a nonlinear least-squares method for the local estimation of Rayleigh wave speed [Fig. 2(d)]. For a semi-infinite homogeneous sample, the shear wave speed can be determined using the following equation $C_{s}=\frac{1+\nu }{0.87+1.12\nu }C_{R}$, where ν is Poisson’s ratio. Thus, measurement of the Rayleigh wave speed gives direct and quantitative access to the shear wave speed.Finally, the aforementioned procedure was repeated by moving the window through the entire surface frame to generate a shear wave speed map along with its probability density distribution. Subsequently, the mean and standard error of the elastic modulus were computed accordingly [Fig. 2(e)]. Opting for a smaller 2D auto-correlation window size would enhance resolution; however, employing a larger window size could potentially yield more accurate estimations of shear wave speeds.^36^ A previous study has suggested that to ensure precise shear wave speed estimation, the window size must cover at least one wavelength.^40^ In the current study, a window size of 1.5 times the shear wavelength was selected based on a trade-off between spatial resolution and estimation accuracy, as smaller windows may introduce noise while larger windows reduce sensitivity to local variations.

### 2.3 Numerical Reverberant Wave Elastography Based on 3D Finite Element Analysis

Numerical simulations were conducted using the 3D finite element method (FEM) software ABAQUS/CAE (Dassault Systèmes Simulia, USA). The phantom’s density ρ and Poisson’s ratio ν were set to $1000\;\mathrm{kg}/m^{3}$ and 0.49, respectively. The Young’s modulus of the phantom was chosen as either 6.75, 3, or 0.75 kPa. Both the Petri dish and scatterers were assumed to have a Young’s modulus of 300 kPa. To ensure accurate simulation of wave propagation, the FEM mesh size was selected to be smaller than one-tenth of the wavelength.

To generate the reverberant displacement field in the phantom surface, multiple methods were employed, and the comparative analysis was carried out. The methods include (1) circle loading (the cycle loading applied on the outer circle of the culture disk bottom); (2) eight-point loading (vibration applied at eight evenly distributed points on the outer circle of the culture disk bottom); (3) sixteen-point point loading (vibration applied at sixteen evenly distributed points on the outer circle of the culture disk bottom); (4) circle loading with eight scatterers (eight scatterers randomly distributed on the outer side in the phantom); (5) circle loading with 16 scatterers; (6) circle loading with 32 scatterers; and (7) circle loading with 64 scatterers. The sine wave motion was applied to the sample with a maximum displacement of 0.1 mm. The particle velocity motion data V(x,y,t) in the surface plane were obtained from the numerical simulation results. The simulation time for samples with theoretical shear wave speeds of 0.5, 1, and 1.5  m/s is 50, 25, and 17 ms, respectively. As a result, the shear wave propagation distance is about 0.75 times the diameter of the culture disk. Numerical simulation is performed in samples to obtain the time-domain displacement field, and the shear wave speed is estimated according to the reverberant wave theory.

### 2.4 Experimental Reverberant Wave Elastography Based on OCT

The reverberant wave elastography system comprises an excitation source and an OCT system.^35^ In addition, the data acquisition card ensures time synchronization among the modulation waveform, mechanical shaker, OCT beam position scan, and OCT data acquisition.

In this study, a novel and straightforward method was employed to achieve the reverberant displacement field within the sample. The excitation setup comprised a function generator, a mechanical shaker (V201, BRUEL & KJAER, Denmark), and 3D-printed randomly distributed scatterers (Fig. 1). Circle loading was applied at the outer circumference of the culture disk bottom. A sinusoidal signal generated by the function generator drove the vibrator.

To measure the displacement field, each acquisition by OCT is composed of a dataset with dimensions (x,y,z,t), where z is the imaging depth, x is the index for points in the lateral direction, y is the index for the elevational direction, and t is the index for the M-scan acquisitions. The particle velocity at the sample surface V(x,y,t) was obtained using the algorithm developed by Loupas et al.^41^ A total of 80 discrete positions (about 5 times the pre-supposed wavelength) were measured in both the lateral, x, and elevational, y directions. Each M-scan obtains the data at 500 axial scans at a 10 kHz scan rate at each position, providing a 50 ms recording for recording wave propagations. Hence, the total acquisition time is about 6 min. To enhance the curve fitting accuracy, 16 points were measured at a distance of one wavelength. Hence, the field of view (FOV) is about 5 times the shear wavelength.

### 2.5 Shear Rheology Study

The hydrogels were subjected to shear rheology tests to measure the storage modulus G′ and loss modulus G′′. A frequency sweep was performed for each sample, ranging from 0.1 to 100  rad/s oscillatory angle frequency. The shear wave speed was determined as a function of angular frequency by utilizing the measured storage and loss modulus at different frequencies,^42^ namely

$$c_{s}(\omega )=\sqrt{\frac{2(G^{\prime }(\omega {)}^{2}+G^{\prime \prime }(\omega {)}^{2})}{\rho (G^{\prime }(\omega )+\sqrt{G^{\prime }(\omega {)}^{2}+G^{\prime \prime }(\omega {)}^{2}})}},$$ (2)

where $c_{s}$ is the shear wave speed, ρ is the sample density, and ω is the angular frequency. The shear wave speed measured from shear rheology will be used to validate that obtained from the OCT reverberant wave elastography.

## 3 Results

### 3.1 Comparison of Reverberant Displacement Pattern Under Varied Loading Conditions

**Fig. 3:**
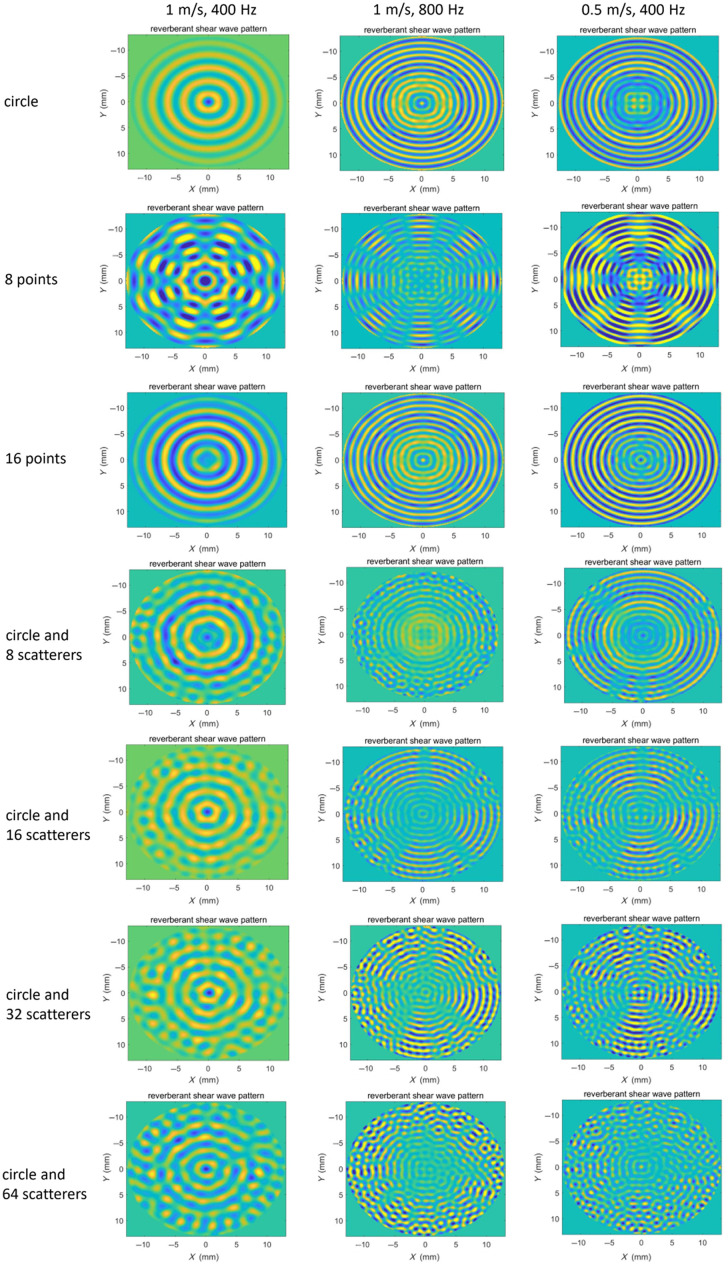
Comparison of the reverberant wave pattern under different loading conditions using numerical simulation.

The theoretical foundation of reverberant wave elastography relies on the establishment of a diffuse wave field, which should satisfy two essential criteria: spatial homogeneity of the wave energy density distribution and directional isotropy of wave propagation components. In this study, we compared reverberant displacement patterns under various loading conditions through finite element simulations. The analysis procedure consisted of three steps: (1) acquisition of time-domain displacement fields under different loading conditions, (2) transformation of the time-domain displacement fields into the frequency domain using fast Fourier transform, and (3) comparative analysis of the resulting frequency-domain displacement fields (Fig. 3).

The frequency-domain displacement fields (Fig. 3) under both circular and 16-point loading configurations display distinct concentric patterns, lacking reverberation characteristics. Although eight-point loading generally produces non-reverberant displacement fields, weak reverberation characteristics emerge only at the maximum wavelength condition (theoretical shear wave speed 1  m/s and loading frequency 400 Hz). Under circular loading with multiple randomly distributed scatterers, a clearly observable reverberation displacement field was generated, and the reverberation characteristics became increasingly prominent with higher scatterer numbers. In addition, it should be noted that the reverberant characteristics become less distinct when the shear wave wavelength is either too large or too small.

**Fig. 4:**
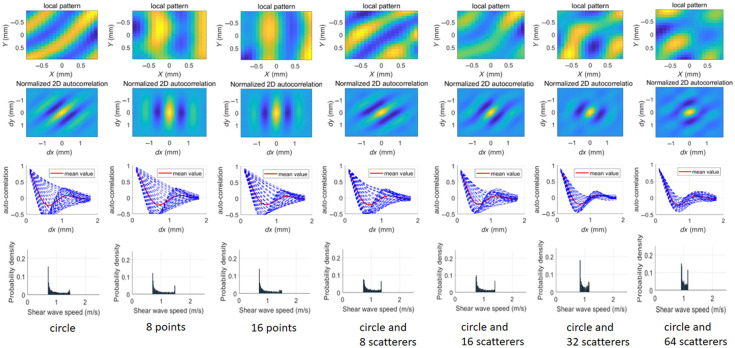
Comparison of the local reverberant wave pattern, normalized 2D auto-correlation map, auto-correlation curves (N=72 curves) taken along radial cuts of the 2D auto-correlation map and their mean value, and the probability density of shear wave speed, under different loading conditions using numerical simulation. Theoretical shear wave speed is 1  m/s, and the loading frequency is 800 Hz.

Figure 4 presents a comparative analysis of local reverberant wave patterns and their corresponding autocorrelation curves under various loading conditions, with a theoretical shear wave speed of 1 m/s and an excitation frequency of 800 Hz. The circular, 8-point, and 16-point loading configurations exhibit wave patterns with obviously directional preferences. By contrast, the circular loading with random scatterers produces isotropic reverberation characteristics, particularly at 64 scatterers. Furthermore, the normalized 2D auto-correlation map and the auto-correlation curves (N=72 curves) taken along radial cuts of the 2D auto-correlation map are compared in Fig. 4. The auto-correlation curves obtained under circular loading, eight-point loading, and 16-point loading deviate significantly from the mean value. By contrast, under circular loading with randomly distributed scatterers, the auto-correlation curves align more closely with the mean value. Notably, as the number of scatterers increases, the curves converge more closely to the mean value. In addition, the Rayleigh wave speeds $C_{R}$ were obtained through curve fitting analysis of the auto-correlation curves (N=72 curves), and the probability density of shear wave speed $C_{s}=\frac{1+\nu }{0.87+1.12\nu }C_{R}$ was shown in Fig. 4. The estimated shear wave speed is closer to the theoretical value with an increasing number of scatterers.

Based on the comparison of reverberant displacement patterns under various loading conditions, circular loading with 64 scatterers was selected for subsequent investigations. This configuration was used in both numerical and experimental reverberation wave elastography performed on the samples.

### 3.2 Reverberant Wave Elastography Using Numerical Simulation

**Fig. 5:**
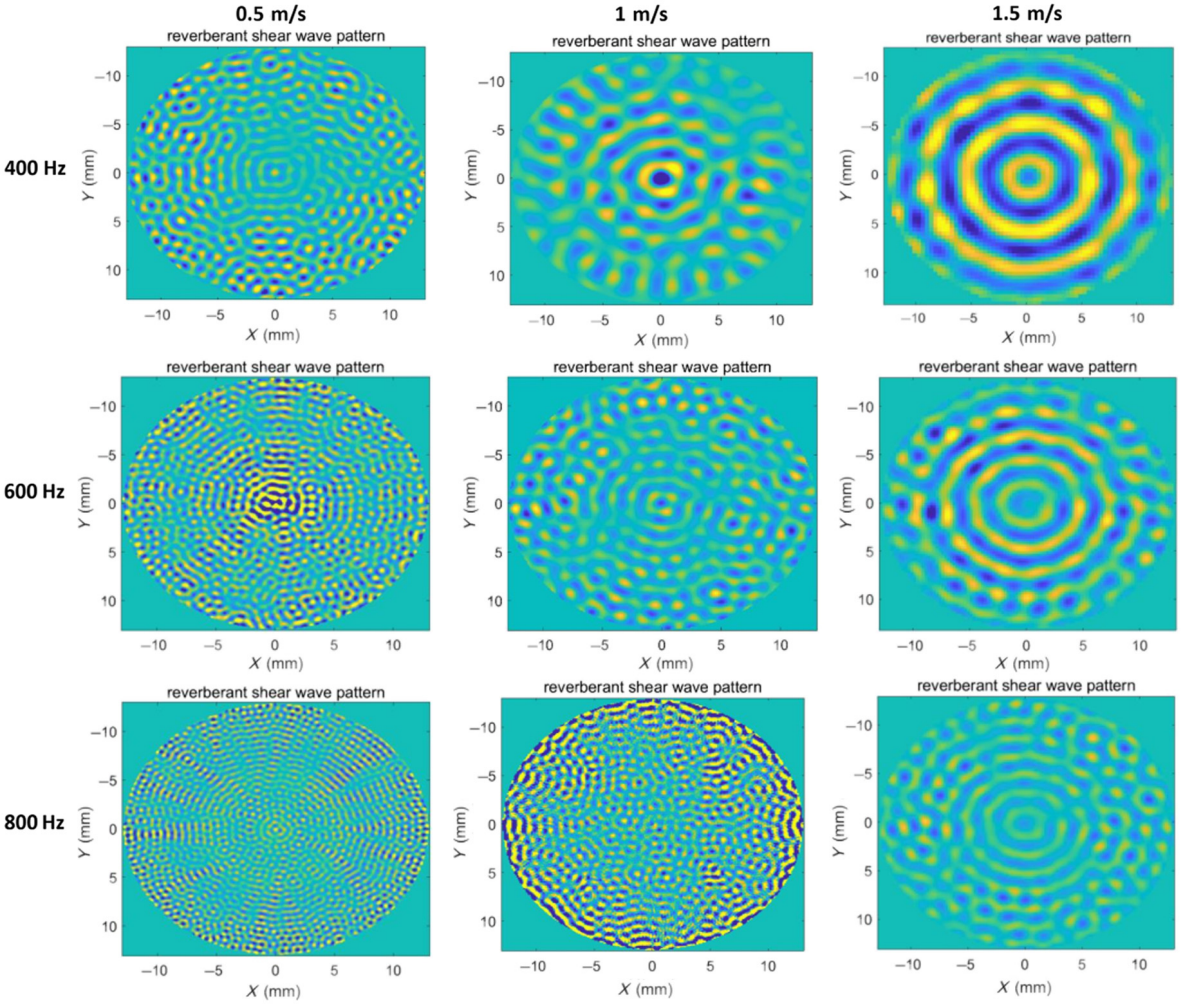
Reverberant wave pattern under loading frequencies of 400, 600, and 800 Hz using numerical simulation. The theoretical shear wave speeds are 0.5, 1, and 1.5  m/s.

Before conducting experimental tests, the feasibility of elasticity measurement using reverberant wave elastography was validated through numerical simulation. First, numerical simulations were performed in samples with shear wave speeds of 0.5, 1, and 1.5  m/s, and with loading frequencies of 200, 400, 600, 800, and 1000 Hz. From these simulations, we acquired the complete time-dependent displacement fields. Subsequently, the fast Fourier transform was applied to the time-domain displacement field to obtain a frequency-domain displacement field (Fig. 5). The numerical simulations revealed distinct reverberation characteristics across different shear wave velocities and excitation frequencies. In samples with a theoretical shear wave speed of 0.5  m/s, the displacement field exhibited well-defined reverberant patterns at loading frequencies of 400 and 600 Hz, although the pattern degraded at 800 Hz. Similarly, for samples with 1  m/s shear wave speed, clear reverberant patterns emerged at 600 and 800 Hz, with some degradation observed at 400 Hz. For samples with 1.5  m/s shear wave speed, optimal reverberant patterns emerged at 800 Hz, with some degradation observed at both 400 and 600 Hz. Hence, the reverberant characteristics become less distinct when the shear wave wavelength is either too large or too small.

**Fig. 6:**
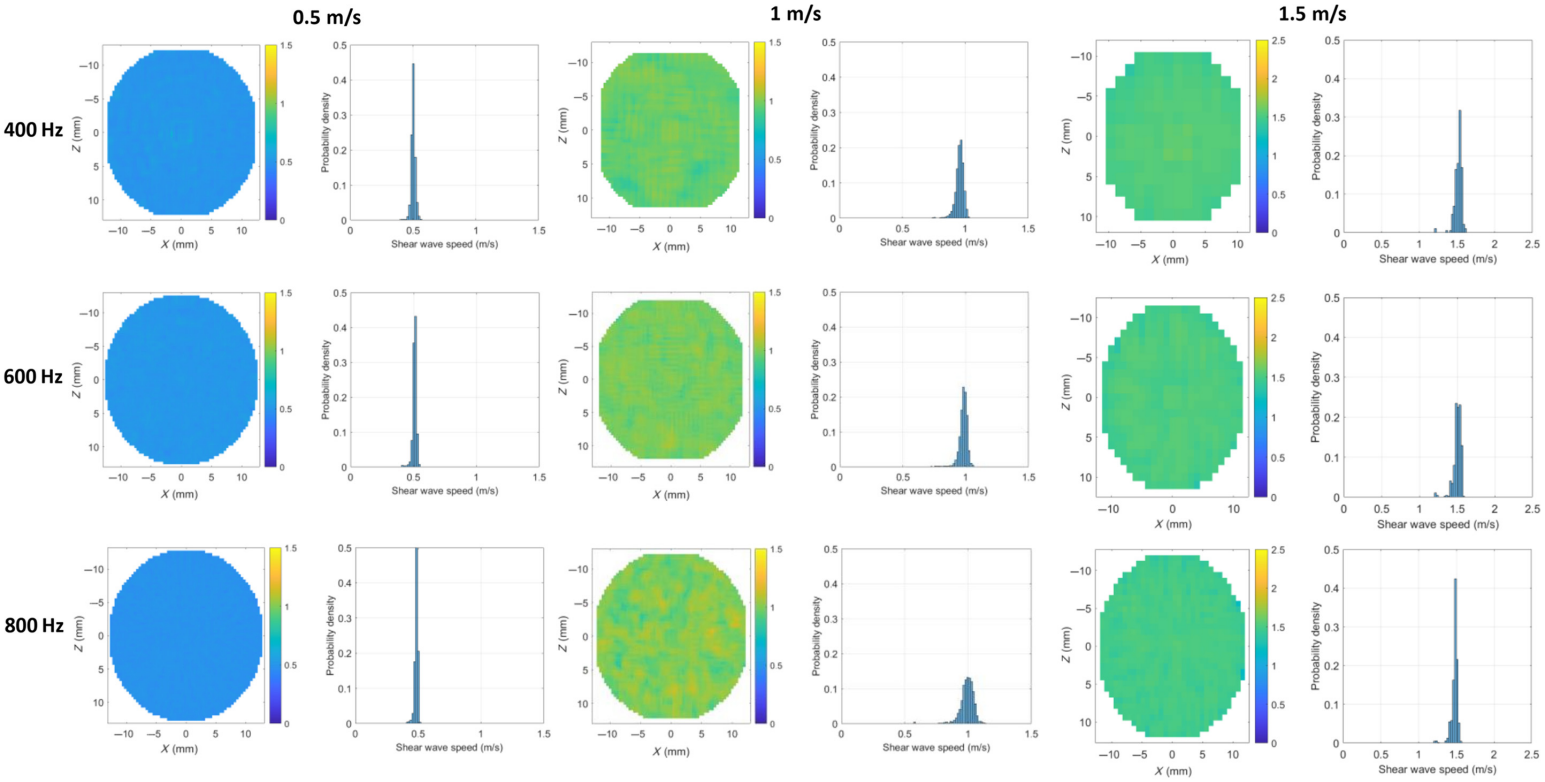
Shear wave speed map and its probability density under loading frequencies of 400, 600, and 800 Hz using numerical simulation. The theoretical shear wave speeds are 0.5, 1, and 1.5  m/s.

**Fig. 7:**
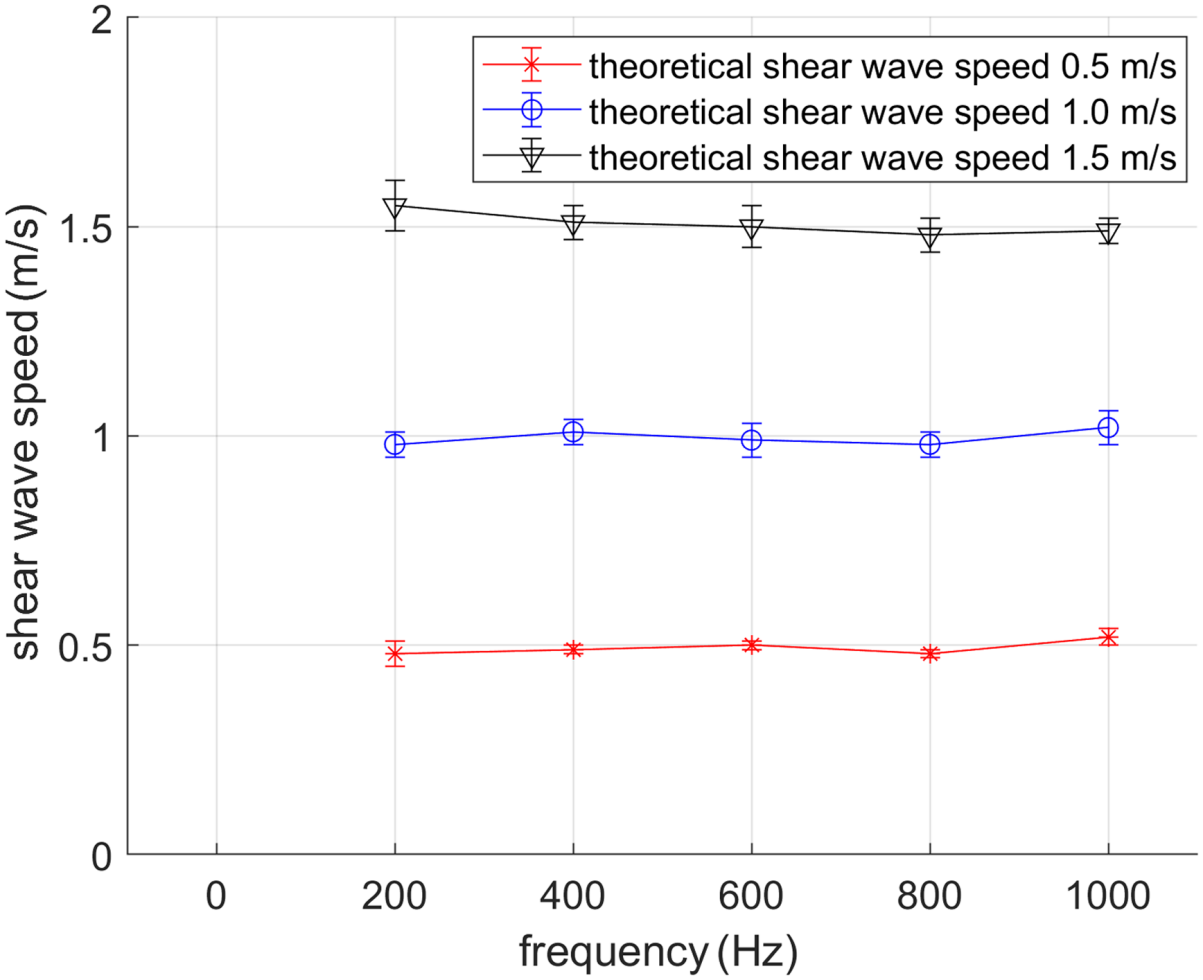
Shear wave speed obtained from the current method using numerical simulation. The theoretical shear wave speeds are 0.5, 1, and 1.5  m/s.

From the frequency-domain displacement field, we calculated the normalized two-dimensional spatial autocorrelation function of the local displacement field patterns. These correlation functions were then used to estimate the Rayleigh wave speed $C_{R}$ based on the reverberant wave theory. Figure 6 presents the shear wave speed $C_{s}=\frac{1+\nu }{0.87+1.12\nu }C_{R}$ maps and their corresponding probability density distributions for phantoms with theoretical shear wave speeds of 0.5, 1, and 1.5  m/s. As demonstrated in Fig. 7, the shear wave speeds estimated through numerical reverberant wave elastography show excellent agreement with the theoretical values. These results confirm the feasibility of implementing reverberant wave-based elasticity reconstruction in culture disk applications.

### 3.3 Reverberant Wave Elastography Using OCT and Shear Rheology Tests

**Fig. 8:**
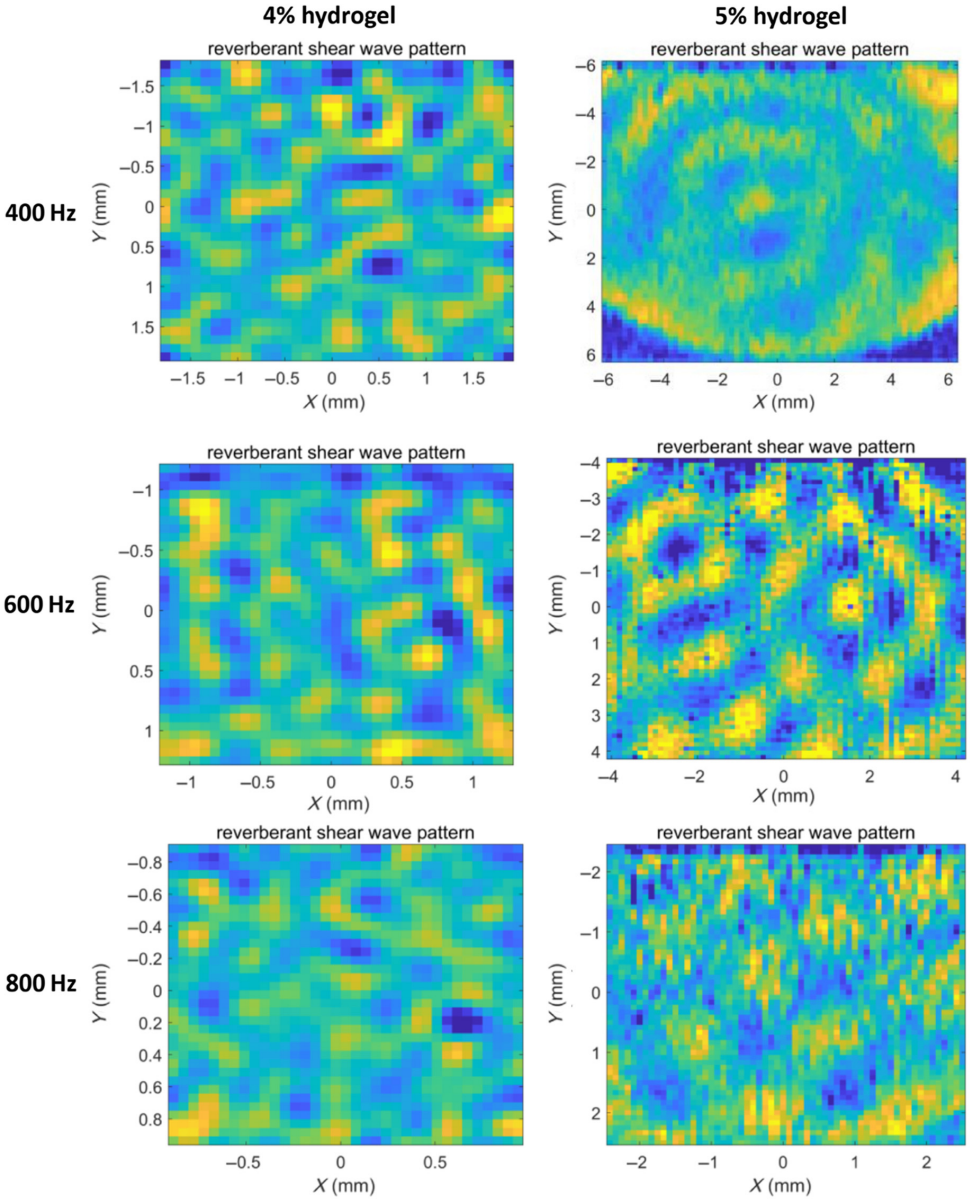
Reverberant wave pattern in samples with hydrogel concentrations 4% v/v and 5% v/v, under loading frequency 400, 600, and 800 Hz.

Elasticity measurement of hydrogel phantoms with hydrogel concentrations of 4% v/v and 5% v/v was performed using reverberant optical coherence elastography. First, the surface time-domain displacement fields were measured using OCT, under loading frequencies of 400, 600, and 800 Hz. A total of 80 discrete positions (about 5 times the pre-supposed wavelength) were measured in both the lateral, x, and elevational, y directions. Then, the fast Fourier transform was applied to the time-domain displacement field resulting in a frequency-domain displacement field (Fig. 8). The displacement fields in 4% v/v hydrogel samples exhibited well-defined reverberant patterns across all tested frequencies (400 to 800 Hz). By contrast, the displacement fields in 5% v/v hydrogels maintained clear reverberant patterns at higher frequencies (600 to 800 Hz) but showed weak reverberant patterns at 400 Hz. That is, when the shear wave wavelength falls outside an optimal range, the reverberant characteristics become less distinct.

**Fig. 9:**
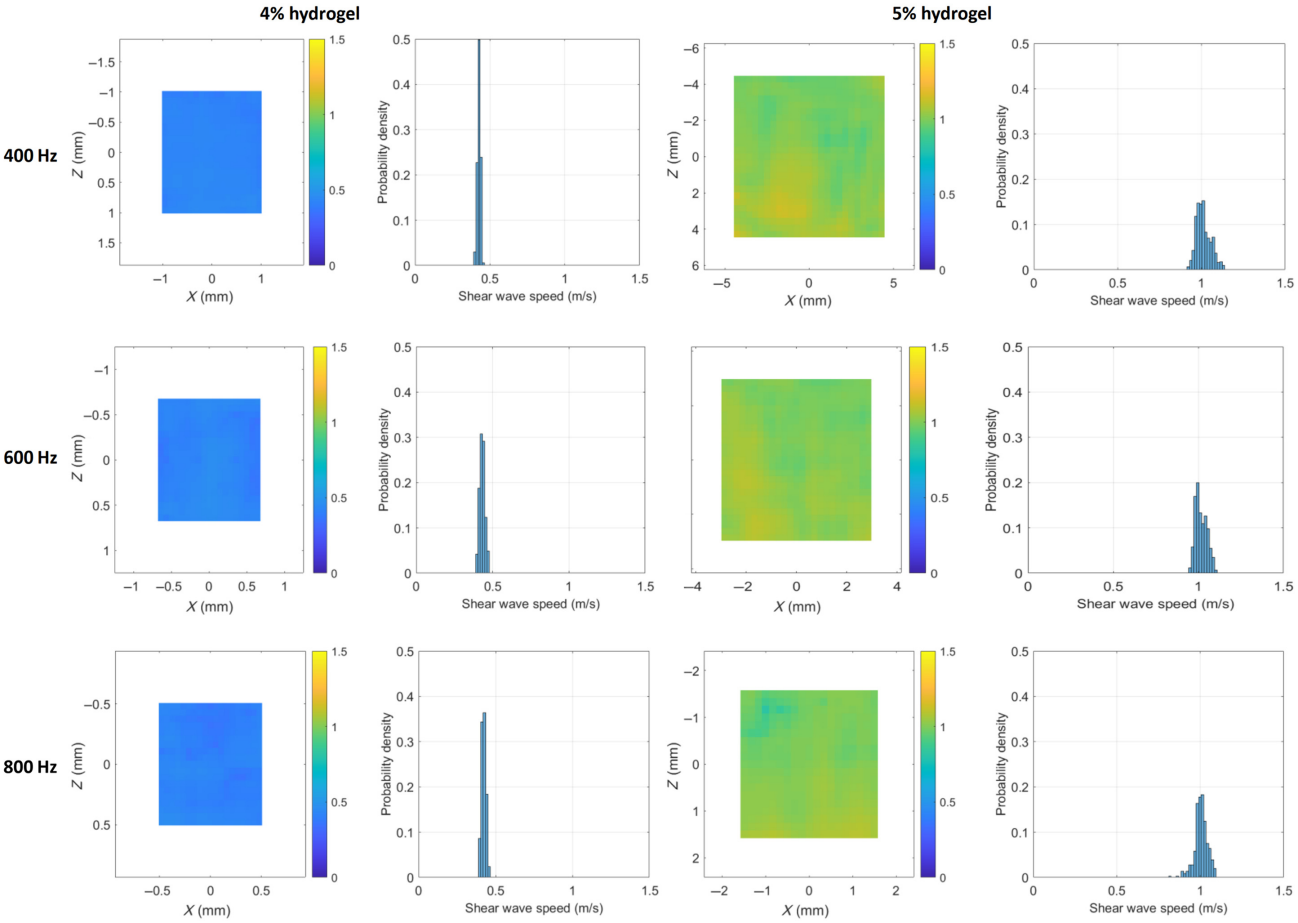
Shear wave speed map and its probability density in samples with hydrogel concentrations 4% v/v and 5% v/v, under loading frequency 400, 600, and 800 Hz.

**Fig. 10:**
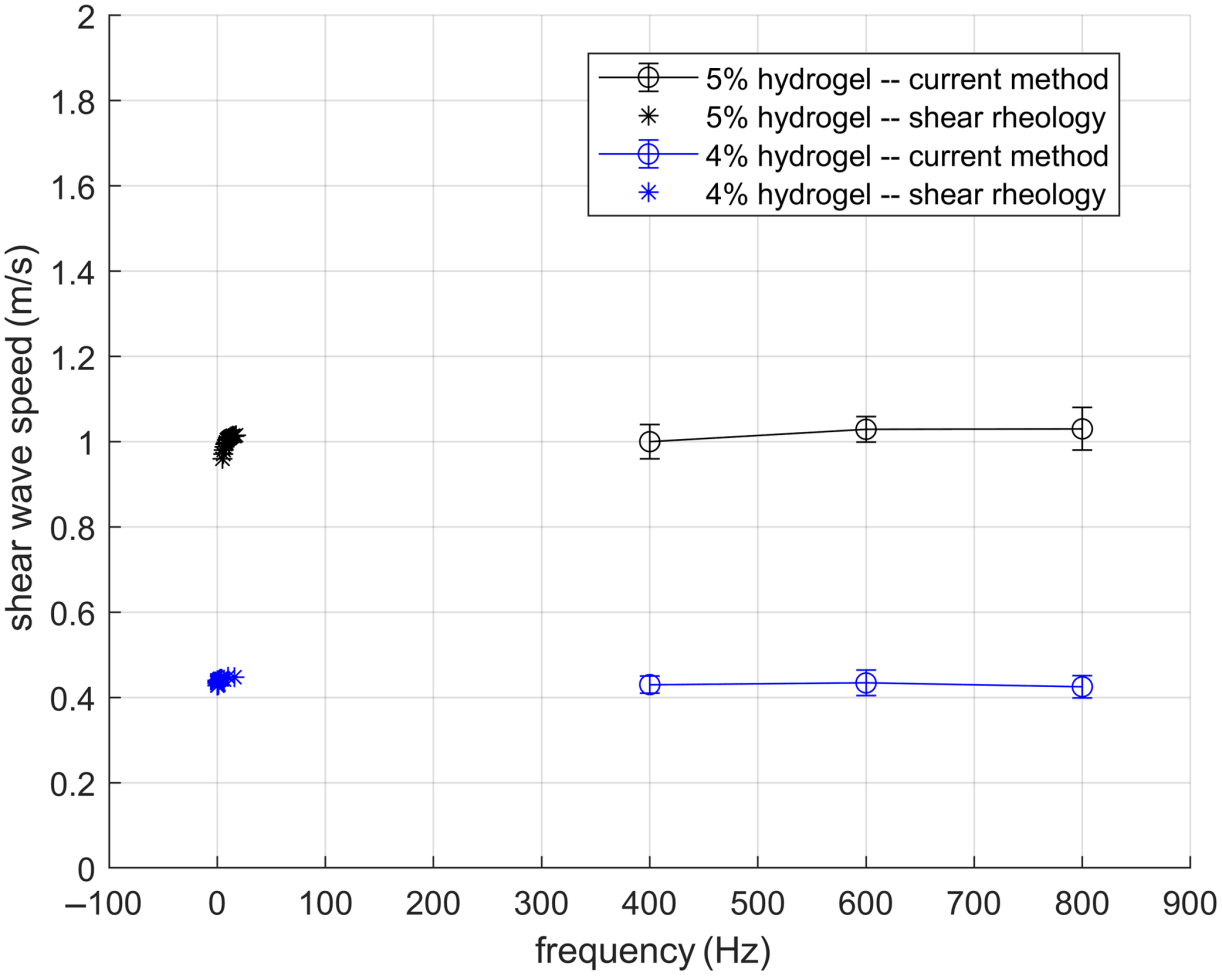
Shear wave speed obtained from the current method and shear rheology method.

From the frequency-domain displacement field data, we computed the normalized 2D spatial autocorrelation map of the displacement patterns. These autocorrelation maps were subsequently analyzed using reverberant wave theory to estimate the Rayleigh wave speed $C_{R}$. Figure 9 presents the shear wave speed $C_{s}=\frac{1+\nu }{0.87+1.12\nu }C_{R}$ maps and their probability distributions for hydrogels at 4% and 5% v/v concentrations. For validation, we performed shear rheology measurements to determine the complex shear moduli of the hydrogel samples, from which theoretical shear wave speeds were calculated using Eq. (2). There is a good agreement between the shear wave speeds obtained from reverberant wave elastography and those from shear rheology tests (Fig. 10). This agreement confirms that reverberant wave method can accurately measure mechanical properties of hydrogel in 3D culture disks.

## 4 Discussion

In reverberant wave elastography, improving the quality of the reverberant displacement field is essential for accurate elasticity estimation. Previous studies have proposed different excitation approaches to generate a reverberant displacement field.^32^^,^^34^^–^^37^ Overall, these methods require a complex device to achieve a multi-point loading. Inspired by a scattering-coded architectural boundary used in object localization,^38^ this study employs 3D-printed randomly distributed scatterers positioned within the culture disk. The current scatterer configuration could significantly improve the quality of the reverberant displacement field, and it is simple and easy to install.

This study compares the reverberation displacement fields generated by regular and random loading distributions. Under regular distribution loading, increasing the number of excitation points degrades the reverberation field quality. This deterioration likely results from the highly periodic spatial excitation pattern, which suppresses the random wave interference necessary for reverberation establishment. By contrast, with randomly distributed scatterers, increasing their number enhances the reverberation displacement field uniformity. These findings demonstrate that the quality of the reverberation field depends not only on the number of loading points or scatterers but also critically on their spatial distribution pattern.

The establishment of a reverberant displacement field requires sufficient wave propagation time to develop diffuse wave events. In corneal tissue (shear wave speed, 0.8 to 2.5  m/s; dimensions, 1 mm thickness × 10 mm diameter), achieving a reverberant state through multi-point excitation requires ∼1  s propagation time.^33^ That is, to achieve the reverberant displacement field, the propagation distance of the shear wave is about 80 to 250 times the cornea diameter. In this study, randomly distributed scatterers were introduced to facilitate reverberant displacement field generation, which significantly reduces the propagation time required for reverberant wave elastography. More specifically, numerical simulations demonstrate that the displacement field can establish a reverberation state when shear waves propagate just 0.75 times the culture disk diameter. Experimental measurements further show that this critical propagation distance is only ∼0.75× and 1.5× the disk diameter for 4% and 5% v/v hydrogels, respectively. Both numerical simulations and experimental measurements confirmed that randomly distributed scatterers enable the rapid establishment of reverberant displacement fields.

The quality of the reverberant displacement field is critically governed by the ratio between shear wavelength (λ) and scatterer size (d). In this study, the shear wavelength ranges from 0.5 to 7.5 mm, whereas the scatterer diameter is maintained at 0.7 mm. This corresponds to a normalized wavelength range of λ/d≈0.71 to 10.7. Both numerical and experimental results reveal that scattering effects become most pronounced when the shear wavelength is comparable to the scatterer diameter, with reverberation characteristics degrading at both very high and very low λ/d ratios. Seismic wave scattering studies have also demonstrated that these scattering phenomena exhibit strong λ/d ratio dependence, maintaining similar scattering behaviour.^43^ Therefore, to generate reverberant displacement fields in samples with a wide range of shear wavelengths (corresponding to different elastic moduli and frequency ranges), we will study randomly distributed scatterers with varying diameters in future research.

The range of excitation frequencies is carefully chosen to ensure that the wavelength is smaller than the phantom thickness, and the field-of-view size is ∼5 times the wavelength. First, at a lower excitation frequency, the wavelength is larger than the sample thickness, causing the wave propagation in the sample to follow the guide wave mode rather than the Rayleigh wave mode, giving rise to more complicated wave behaviour. Second, the field-of-view size is selected to be ∼5 times the wavelength, inducing a more accurate estimation of shear wave speed. Future research will involve the exploration of elasticity measurements at a lower excitation frequency.

The elastic properties are currently estimated by curve fitting the autocorrelation function, but this approach requires an auto-correlation windowing that inherently limits the spatial resolution of the shear wave speed maps. To address this limitation, we will implement the reverberant phase gradient method for wave speed estimation,^44^ which exploits phase information in the displacement field to potentially overcome the spatial resolution constraints imposed by autocorrelation windowing.

In the current methodology, shear wave speed measurements are performed by applying single-frequency harmonic excitations across a 400 to 800 Hz range, which is inherently time-consuming. To enhance measurement efficiency and facilitate practical applications, multi-frequency excitation protocols^40^ will be employed to better capture the hydrogel’s complete viscoelastic response.

## 5 Conclusion

This study employed 3D-printed randomly distributed scatterers in reverberant wave elastography for shear wave speed estimation in hydrogel phantoms. First, numerical simulations were conducted to validate the advantages of these scatterers, demonstrating their effectiveness in significantly improving the quality of the reverberant displacement field. Building upon these findings, numerical reverberant wave elastography was successfully implemented, confirming the feasibility of elasticity measurements for samples in culture dishes. Experimental validation revealed excellent agreement between the proposed method and conventional shear rheology tests, highlighting its potential as a reliable and valuable approach for characterizing the mechanical properties of soft biomaterials.

## Data Availability

The original contributions presented in the study are included in the article, and further inquiries can be directed to the corresponding author.
